# Efficacy of *Ficus sycomorus* (Sycamore Fig) Extract on Intestinal Coccidiosis in Experimentally Infected Rabbits

**DOI:** 10.3390/life12060917

**Published:** 2022-06-18

**Authors:** Ahmed Dawod, Said Fathalla, Hesham R. El-Seedi, Mohamed A. Hammad, Noha Osman, Nagwa Abosheriba, Anis Anis, Awad A. Shehata, Ahmed Elkhatam

**Affiliations:** 1 Department of Husbandry and Animal Wealth Development, Faculty of Veterinary Medicine, University of Sadat City, Menoufia 32897, Egypt; adawod@vet.usc.edu.eg (A.D.); noha.osman@vet.usc.edu.eg (N.O.); nagwa.abosheriba@vet.usc.edu.eg (N.A.); 2 Department of Physiology, Faculty of Veterinary Medicine, University of Sadat City, Sadat City 32897, Egypt; saidfathalla@vet.usc.edu.eg; 3 Pharmacognosy Group, Department of Pharmaceutical Biosciences, Biomedical Centre, Uppsala University, P.O. Box 591, SE 751 24 Uppsala, Sweden; hesham.el-seedi@farmbio.uu.se; 4 International Research Center for Food Nutrition and Safety, Jiangsu University, Zhenjiang 212013, China; 5 Department of Chemistry, Faculty of Science, Menoufia University, Shebin El-Kom 32512, Egypt; 6 Department of Analytical Chemistry, Faculty of Pharmacy, University of Sadat City, Sadat City 32897, Egypt; m_abdelkhalek_eg@fop.usc.edu.eg; 7 Department of Pathology, Faculty of Veterinary Medicine, University of Sadat City, Sadat City 32897, Egypt; aniszaid@vet.usc.edu.eg; 8 Avian and Rabbit Diseases Department, Faculty of Veterinary Medicine, University of Sadat City, Sadat City 32897, Egypt; 9 Research and Development Section, PerNaturam GmbH, 56290 Gödenroth, Germany; 10 Prophy-Institute for Applied Prophylaxis, 59159 Bönen, Germany; 11 Department of Parasitology, Faculty of Veterinary Medicine, University of Sadat City, Sadat City 32897, Egypt; ahmed.osman@vet.usc.edu.eg

**Keywords:** *Eimeria intestinalis*, rabbits, *Ficus sycomorus*, coccidia, rabbits

## Abstract

This study was conducted to investigate the effect of the *Ficus sycomorus* extract on *Eimeria intestinalis* in experimentally infected rabbits. For this purpose, forty male 30-day-old rabbits (*Blanc de Bouscat*) were divided into four groups (*n* = 10 in each group). Rabbits kept in the first group served as negative control (non-treated-non-infected). Rabbits kept in the second, third, and fourth groups were challenged at 10 weeks old with 3 × 10^4^ *E. intestinalis* sporulated oocysts. The third and fourth groups were treated orally with diclazuril 10% (0.05 mg/kg body weight) and *F. sycomorus* (100 mg/Kg) for three consecutive days, respectively. The efficacy was assessed based on the growth performance parameters, clinical symptoms, oocyst shedding, histopathological findings, and hematological parameters for 16 days post challenge. The study revealed that rabbits treated with *F. sycomorus* methanolic extract and diclazuril showed mild clinical symptoms with a significant decrease in oocyst shedding compared with the positive control. Moreover, the diclazuril-treated group showed the highest leukocytic count and the lowest monocytes percentage compared with other groups. Furthermore, the lowest lymphocytes percentage was recorded in the control positive group. Histopathologically, moderate coccidia infestation in the intestinal mucosa and moderate hydropic degeneration of hepatocytes were observed in the diclazuril treated group compared with the negative control. However, mild coccidia infestation in the intestinal mucosa and slight coagulative necrosis of hepatocytes was found in the *F. sycomorus* treated group. In conclusion, *F. sycomorus* methanolic extract had promising effects on the live performance, oocyst count, and blood variables, while it possesses adverse consequences on the hepatic tissues. Further studies are required to optimize the dose and extraction method to mitigate its side effects.

## 1. Introduction

Rabbit coccidiosis, caused by *Eimeria* spp., is a highly contagious protozoal disease affecting rabbits. This disease is considered a serious health hazard associated with massive economic and productive losses worldwide [1,2]. *Eimeria* spp. can negatively impact the digestive system function by destroying the intestinal epithelial cells and bile ducts, which may result in many digestive disorders such as bloat, diarrhea, and intoxication [3]. Coccidiosis in rabbits has two main common forms, namely intestinal and hepatic. Fourteen species of the genus *Eimeria* have been recognized as pathogens of rabbit coccidiosis. Thirteen of them infect the gastrointestinal tract causing intestinal coccidiosis, while *E. stiedae* infects the endothelial cells of the bile ducts and causes hepatic coccidiosis [4]. *E. intestinalis* is one of the highly pathogenic and immunogenic species [5]. This disease is known to cause severe clinical symptoms, such as depression, low weight gain, diarrhea, and mortality [6]. Young rabbits of 1–3 months old are more susceptible to coccidiosis, especially during their weaning stage [3]. The maximum excretion of *Eimeria* oocysts in growing rabbits appeared around the age of 50 days, then considerably declined by the age of 81 to 109 days [7]. In contrast, adult rabbits had low *Eimeria* oocysts excreted in the feces, while it may be higher in does during the period from delivery till 18–25 days post-partum [7,8].

Coccidia is highly resistant to the most commonly used disinfectants. This nature makes the total eradication of such protozoa from rabbit farms completely impossible [3]. Therefore, it is recommended to use coccidiostats in rabbit’s diet or drinking water to alleviate the negative effects of coccidiosis [9]. However, the long-term use of coccidiostats has been shown to result in a significant coccidiostat resistance in rabbit flocks [10]. Many cases of spreading of robenidine-resistant strains of *E. magna*, *E. media*, and *E. perforans* had been reported on rabbit farms [3,11]. Previously, it has been reported that coccidiostat used for controlling of coccidiosis was also detected in edible animal products [12]. Consequently, there is an urgent need to develop new alternative strategies for controlling and preventing rabbit coccidiosis. Tannins-rich plants are promising strategy for controlling coccidiosis in lambs [13,14] and kids [15]. In addition, feeding rabbits on diets containing sainfoin (*Onobrychis viciifoliae*), rich in tannins, can succeed in reducing the fecal oocyst output of *Eimeria* spp. and improving the feed conversion ratio [16]. 

*Ficus sycomorus (F. sycomorus)* is also a tannins-rich plant belonging to the mulberry family (family *Moraceae).* The value of condensed tannins reached 46% in the stem bark extract of *F. sycomorus,* which mainly consisted of procyanidin-type tannins termed quercetin [17]. Quercetin has antiprotozoal and antifungal effects in humans [18]. In addition, *F. sycomorus* stem bark extract contains chlorogenic acid [19], which exhibits antibacterial [20], antioxidant [21,22,23], and anti-inflammatory [21] effects. Dietary supplementation of chlorogenic acid in chickens suffering from necrotic enteritis resulted in depression of proinflammatory mediators’ expression, such as interferon (IFN)-β, IFN-γ, interleukin-1 (IL-1), IL-17A, IL-22, and tumor necrosis factor-α, with improvement of the growth performance [21,24]. Other pharmacologic properties of *F. sycomorus* such as antibacterial [25,26], antifungal [27], and trypanocidal activities [28] have been reported. Additionally, several researchers have reported the significant effects of *F. sycomorus* in rats and mice such as antioxidant [29], hepatoprotective [30], anti-inflammatory [31], and antidiarrheal agent [32].

It is well-documented that coccidiosis infection results in severe oxidative stress and inflammatory reaction in many species, including rabbits [33]. Thus, the anti-inflammatory and antioxidant activities of *F. sycomorus* may result in a rapid amelioration of the negative effects of coccidiosis in rabbits. Moreover, chlorogenic acid exhibited an anticoccidial effect and an improvement of intestinal barrier function in coccidia-infected broilers [21]. The current study aimed to investigate the potential anticoccidial effect of the methanolic extract of *F. sycomorus* stem bark on controlling intestinal coccidiosis in growing rabbits, with emphasis on *E. intestinalis* because of its wide range prevalence and pathogenicity. Moreover, the consequences on rabbit growth performance were also studied. 

## 2. Materials and Methods

### 2.1. Ethical Statement 

All experimental procedures of this study have been approved by the “Animal Ethics Committee” of the University of Sadat City, Egypt.

### 2.2. Plant Materials and Preparation of the Extracts

**Figure 1:**
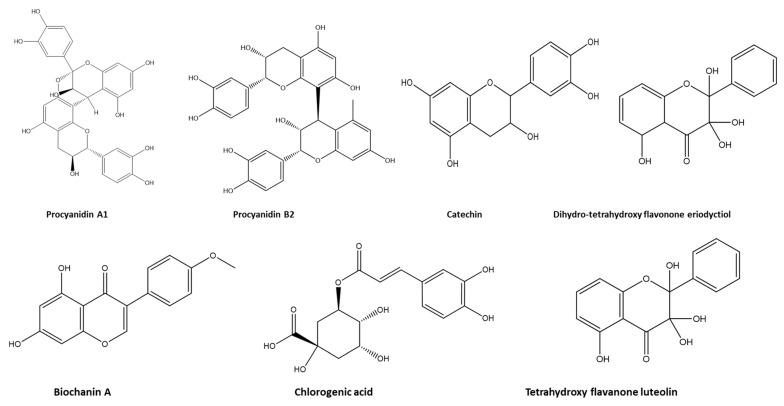
Isolated and identified compounds from methanolic extract of *F. sycomorus*.

The bark samples of *F. sycomorus* were obtained from Menofia Governorate, Egypt. The samples, collected at 5 a.m. on 24 March 2020, were tightly packaged in plastic bags. Then, they were air-dried and ground using a Thomas–Willey milling machine of 60-mesh size. The extraction was performed by soaking 100 g of the grounded *F. sycomorus* stem bark in 500 mL of 70% methanol for 3 successive days. The mixture was filtered and dried with a rotatory evaporator under reduced pressure at 22 °C till the desired weight was achieved. The final product was stored in a dark-brown glass bottle at 4 °C till further use. In our previous study, the extract was subjected to high-resolution mass spectrometric measurements via ultra-high performance liquid chromatography-quadrupole time-of-flight-nanospray mass spectrometry (UPLC-QToF-MS) and 1H NMR analysis [19]. The chemical structure of the identified molecules is shown in Figure 1.

### 2.3. Rabbits

Forty newly weaned male rabbits belonging to the *Blanc de Bouscat* breed were selected from a local governmental farm in the Sharqia Governorate, Egypt. Rabbits were approximately one month old, and the average body weight was 0.714 ± 0.07 kg. They were housed in individual galvanized wire cages under coccidia-free conditions with a separate waterspout and feeder. Rabbits were fed coccidia-free pellets once a day, and water was available ad libitum. The gross composition (%) of the concentrate diet is shown in Table 1. 

The coccidia-free condition was obtained via heating of the feeding pellets and/or drinking water to 80 °C for 2 h before offering to the rabbits. The animals were monitored during an adjustment period of 8 weeks before the initiation of infection. The adjustment period allowed the rabbits to be dewormed and acclimated to pen living and routine feeding. The newly weaned rabbits were kept in a coccidia-free environment and were delivered from breeding stock routinely dose with diclazuril 1 mg/L in drinking water [34]. Fecal samples were examined weekly for the presence of coccidia oocysts to confirm the coccidia-free cases. In the third week of the adjustment period, all the animals were dewormed with fenbendazole (4 mL/100 Kg BW).

### 2.4. Sporulated Oocyst 

The sporulated *E. intestinalis* oocysts were obtained from the laboratory of the Parasitology Department, Faculty of Veterinary Medicine, University of Sadat City, Egypt. The oocysts were collected from the feces of naturally infected rabbits. A pure strain of *E. intestinalis* was obtained by single-oocyst isolation [35], propagated, and preserved according to established protocols [36]. Briefly, the oocysts were stored in a solution of 2.5% potassium dichromate for efficient oocyst sporulation. Immediately before infection initiation, the sporulated oocysts were washed to remove the potassium dichromate solution and adjusted in standard saline solution to 3 × 10^4^ oocysts per mL.

### 2.5. Experimental Design

Starting on week 10, rabbits were randomly assigned into four experimental groups, with ten animals per group. Groups 2, 3, and 4 were inoculated via oral gavage with 3 × 10^4^ of *E. intestinalis* oocysts. Group 1 was kept in a separate room under the same experimental conditions and received no coccidia oocysts or treatment and served as the unchallenged negative control group (−ve control). Fecal samples were collected from inoculated animals individually in each group daily till the coccidia infection was confirmed. After confirmation of infection in the inoculated groups, the second group received no treatment therapy and served as the challenged, untreated positive control group (+ve control), while the third group received diclazuril 10% (0.05 mg/Kg BW) at 4th day post challenge orally dosed for three consecutive days and served as challenged, diclazuril-treated group (diclazuril group). The fourth group received *F. sycomorus* extract at the dose rate of 100 mg/kg BW for four consecutive days and served as the challenged and extract-treated group (extract group). The experimental design is shown in Table 2. 

### 2.6. Fecal Oocyst Examination and Clinical Presentation of Coccidiosis

Fecal samples were collected daily, starting from the 4th to the 16th day post challenge. Total coccidia oocyst output was determined for each animal in each trial using the Mac Master technique [37]. Clinical signs of general depression, diarrhea, and mortality were reported at the 8th day post challenge. 

### 2.7. Liver Function and Hemogram 

Blood samples were withdrawn from the marginal ear vein at the 4th, 8th, 12th, and 16th days post-infection. Then, samples were divided into three parts; the first part was centrifuged at 3000 rpm to obtain the serum, while the second part was heparinized to estimate alanine aminotransferase (ALT), aspartate aminotransferase (AST), and alkaline phosphatase (ALP) via a spectrophotometer (WPA, Biochrom, Cambridge, UK) and measuring kits (Spectra CO. Cairo, Egypt), while the third part was preserved with EDTA to examine the blood erythrogram and leukogram using automated hematology analyzers. 

### 2.8. Histopathological Examination

Three rabbits per each treatment group (12 rabbits total) were randomly selected and subsequently euthanized with sodium pentobarbital anesthesia (60 mg/Kg) at the 8th day post-infection. Tissue samples were taken from the jejunum and liver and fixed in 10% formalin for 72 h. For analysis, the samples were trimmed, washed, dehydrated, and then embedded in paraffin wax. Afterward, the samples were serially sectioned with 3 μm thickness using a microtome (Leica, Wetzlar, Germany) and stained with hematoxylin and eosin (H&E) stain [38]. Additionally, histopathological images were taken using Leica DMLB microscopes and Leica EC3 digital camera for intestinal lesions (mucosal hyperplasia and intensity of coccidia parasite in intestinal mucosa) and liver lesions (hydropic degeneration of hepatocytes, coagulative necrosis of hepatocytes, dilatation and engorgement of blood sinusoids, dilatation and engorgement of bile duct, and presence of fat globules in bloodstream of central veins). The comparative histopathological lesion scoring in different groups was carried out by examining 5 high-power fields (×40), where 0, 1, 2, 3, and 4 scores denoted absence, minimal, mild, moderate, and severe lesions, respectively, in each examined region. The score assigned could be defined as the % of the affected area by the lesion [39,40]. 

### 2.9. Growth Performance 

Body weight, body gain, feed intake, and feed conversion were measured beginning from the day of challenge till day 16 post challenge. 

### 2.10. Statistical Analysis

After the completion of the experiment, the data were statistically analyzed using the SAS software (SAS User’s Guide: Statistics, Version 9.2 Edition, 2008, SAS Inst. Inc., Cary, NC, USA). Data of *E. intestinalis* oocyst, RBCs, and WBCs counts were logarithmically transformed to obtain normally distributed values. Then, the antilogarithm was obtained for each least squares mean after the analysis. Moreover, arcsine transformation was performed for the percentage data of differential leukocyte counts and mean corpuscular hemoglobin concentration (MCHC). Repeated-measure ANOVA with PROC MIXED method of the SAS software was used to analyze the repeated measure data of fecal oocyst output of *E. intestinalis*, liver enzymes, erythrogram, and leukogram using the following statistical model:

$$Y_{ijk}=\mu +\alpha_{i}+d_{j\left(i\right)}+\tau_{k}+{\left(\alpha \tau \right)}_{ik}+\epsilon_{ijk}$$

where $Y_{ijk}$ = overall observation; µ = overall mean; $\alpha_{i}$, $\tau_{k},$ and ${\left(\alpha \tau \right)}_{ik}$ are fixed effects of treatment; *i* = 1 to 4; time *k* = 4th, 8th, 12th, and 16th day post-infection for liver enzymes, erythrogram, and leukogram or 4th, 6th, 8th, 10th, 12th, 14th, and 16th day post-infection for the data of fecal oocyst output of *E. intestinalis*; $d_{j\left(i\right)}$ is the random effect associated with the jth subject in group *i*; $\epsilon_{ijk}$ is the random error associated with the jth subject in group *i* at time *k*. Bonferroni post hoc test was performed as a mean separation test for the repeated-measure analysis. Results were expressed as least squares means ± SE. 

In addition, initial body weight, covariate body weight, gain, feed intake, and feed conversion were analyzed using one-way ANOVA of the linear mixed model with Duncan’s multiple range test as a post hoc test according to the following statistical model:

$$Y_{ij}=µ+\alpha_{i}+\epsilon_{ij}$$

where $Y_{ij}$ = overall observation; µ = overall mean; $\alpha_{i}$ is the effect of treatment *i* = 1 to 4; $\epsilon_{ij}$ is the random error. 

Fisher’s exact test was performed to analyze the coccidiosis symptoms (diarrhea and depression) among the various experimental groups. 

Differences among mean intestinal lesion scores were analyzed using the procedure for nonparametric models using Kruskal–Wallis one-way analysis of variance (PROC NPAR1WAY) and general linear mixed models (PROC MIXED) of SAS 9.2© (SAS Institute, 2008), and Dunn–Bonferroni least square means comparison was used for means separation [41]. Differences among means were deemed significant at *p* ≤ 0.05. 

## 3. Results

### 3.1. Effects of F. sycomorus Extract on Oocyst Shedding

**Figure 2:**
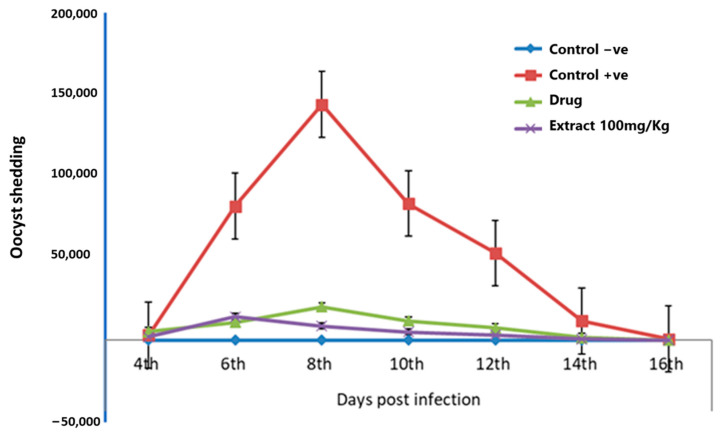
Effects of *F. sycomorus* extract on the shedding on the fecal oocyst output of *E intestinalis* in rabbits through 16 days post challenge. Control −ve, unchallenged and untreated; Control +ve, challenged with *E. intestinalis* and untreated; Drug, challenged with *E. intestinalis* and treated with diclazuril; Extract 100 mg/Kg, challenged with *E. intestinalis* and treated with *F. sycomorus* extract. Rabbits treated with *F. sycomorus* extract showed a significant decrease in oocyst shedding compared with the positive control (*p* < 0.0001).

The *E. intestinalis* oocyst shedding was determined every other day from day 4 to day 16 post-infection (Figure 2). The peak oocyst production was reported on the 8th day of infection in the infected groups. The highest production of the *E. intestinalis* oocyst was attained in the positive control group (143,879.90 ± 0.24 oocyst/g), followed by the diclazuril-treated group (20,384.50 ± 0.34 oocyst/g) and the extract-treated group (8772.03 ± 0.28 oocyst/g). Our findings revealed that the use of an infection dose of 1 × 10^4^ *E. intestinalis* resulted in a maximum oocyst output of 143,879.90 ± 0.24 oocyst/g in the positive control group.

Significant differences in oocyst output were observed among the different experimental groups. Oocyst shedding was first observed in feces at day 4 post challenge in all challenged groups, with the highest count observed at day 8 post challenge. The negative control group recorded zero oocyst shedding throughout the experiment. Rabbits treated with 100 mg/Kg *F. sycomorus* extract showed a significant decline in average oocyst output throughout the experiment (1867.67 ± 0.10 oocyst/g) compared with either diclazuril (3895.83 ± 0.12 oocyst/g) or positive control group (20,749.14 ± 0.09 oocyst/g) (*p* < 0.0001). This trend appeared throughout the experiment from the 4th to the 16th day post challenge (*p* < 0.0001). 

### 3.2. Impact of F. sycomorus Extract on the Clinical Presentation of Coccidiosis

Rabbits belonging to the negative control group exhibited no clinical signs of coccidiosis (Table 3).

Moreover, no mortalities were reported in rabbits belonging to groups 2, 3, and 4. Furthermore, no significant differences were reported among the different groups in the appearance of general depression cases. General depression cases were increased in rabbits belonging to positive control groups (30%), followed by diclazuril (20%) and extract-treated (20%) groups. However, *F. sycomorus* extract significantly depressed diarrhea; out of the 10 coccidia-infected cases treated with 100 mg/Kg of *F. sycomorus* extract, no case exhibited diarrhea (*p* < 0.01).

### 3.3. Effects of F. sycomorus Extract on Liver Function and Hemogram

A significant decrease in the ALT level was detected in the negative control group (27.26 ± 3.55 IU/L), followed by diclazuril (21.10 ± 3.27 IU/L) and extract-treated (20.17 ± 3.29 IU/L) groups compared with the positive control group (34.01 ± 3.55 IU/L) (Table 4).

Furthermore, no significant differences were reported in the AST and ALP levels among the different experimental groups. No significant differences were observed in Hb, RBCs, and MCH values among the groups (Table 4). The values of MCV were significantly decreased in the diclazuril and *F. sycomorus*-treated groups (40.88 ± 0.56; 40.90 ± 0.58 fl, respectively) and positive control group (41.28 ± 0.64 fl) compared to the negative control group (43.81 ± 0.63 fl). In contrast, a significant decrease in MCHC was observed in the negative control group (51.75 ± 1.09%) compared with the diclazuril or *F. sycomorus*-treated groups (56.11 ± 0.98; 56.18 ± 1.02%, respectively). However, the latter two groups did not differ from the positive control group (54.40 ± 1.12%) (*p* < 0.03).

As shown in Table 4, rabbits treated with diclazuril or *F. sycomorus* extract demonstrated a significant increase in the total WBCs count (10.21 ± 0.02 × 10^3^; 9.70 ± 0.03 × 10^3^, respectively) when compared with the negative control group (7.81 ± 0.03 × 10^3^). The positive control group had the lowest lymphocyte percentages (53.83 ± 2.78%), which was statistically significant compared with the other groups. The highest lymphocyte percentage was recorded in the negative control group (70.34 ± 2.71%), followed by diclazuril- and *F. sycomorus* extract-treated groups (61.76 ± 2.44; 55.06 ± 2.52%), where the lowest lymphocytes% was recorded in the control positive group (53.83 ± 2.78%). Furthermore, the diclazuril-treated group record the highest total WBCs (10.21 ± 0.02 × 10^3^) and the lowest monocytes % (9.25 ± 0.73%) compared to the others (*p* < 0.05).

No significant changes were identified in monocyte percentages between the negative and positive control groups. Nevertheless, there was a significant decline in their percentages in the diclazuril- and extract-treated groups (9.25 ± 0.73; 9.37 ± 0.73%, respectively) compared with the control negative group (10.11 ± 0.73%) (*p* < 0.04). Granulocyte percentages were significantly elevated in the positive control group (39.79 ± 2.40%), followed by the diclazuril- or extract-treated groups (33.97 ± 2.10; 39.74 ± 2.17%, respectively), whereas the lowest granulocyte percentages were observed in the negative control group (25.83 ± 2.34%) (*p* < 0.03). 

### 3.4. Pathologically: Medication Tends to Secure Hepato-Intestinal Tissue Architectures

Table 5 presents the histopathological investigation of the intestinal tissue of *E. intestinalis*-infected groups at the 8th day post-infection.

It was found that the positive control group had a higher score for mucosal hyperplasia (3.33 ± 0.33) than the negative control group (0.00 ± 0.00), which was not significantly differed from either diclazuril (2.83 ± 0.17) or extract (3.00 ± 0.00) treated groups (*p* < 0.03). The same trend appeared in the results of the intensity of the coccidia parasite in the intestinal mucosa (*p* < 0.01). In addition, the positive control group attained a higher score of dilatation and engorgement of blood sinusoids (2.83 ± 0.17) compared with the negative control group (0.00 ± 0.00), while it increased the score of dilatation and engorgement of the bile duct (1.67 ± 0.33) more than either the treated or control negative groups (*p* < 0.01). Moreover, the challenged and treated groups recorded higher scores of the presence of fat globules in the bloodstream of central veins (4.00 ± 0.00) compared with either positive (2.33 ± 0.33) or negative control (0.00 ± 0.00) groups.

**Figure 3:**
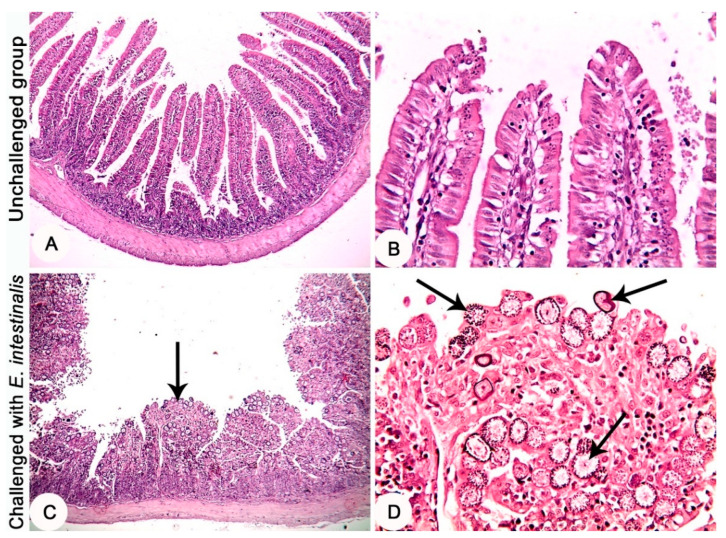
Jejunum of rabbits at the 8th day post challenge with *E. intestinalis*. (**A**,**B**) Negative control group (unchallenged and untreated) showing normal histological architecture. (**C**,**D**) Positive control group (challenged with 3 × 10^4^ *E. intestinalis* oocysts-untreated), (**C**) showing marked hyperplasia of the intestinal mucosa (arrow). (**D**) Showing severe infestation with the developmental stages of *E. intestinalis* in the intestinal mucosa (arrows). HE stain, (**A**,**C**) = ×4; (**B**,**D**) = ×20.

**Figure 4:**
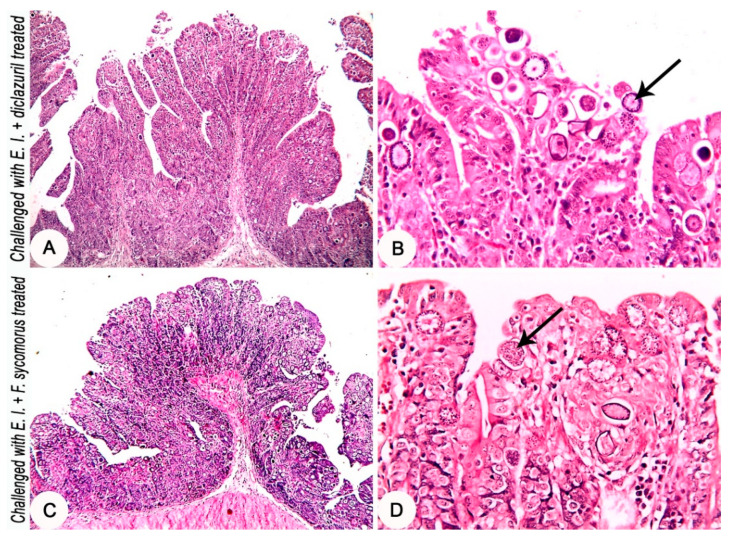
Jejunum of rabbits at the 8th day post challenge with *E. intestinalis*. (**A**,**B**) Diclazuril-treated group (challenged with 3 × 10^4^ *E. intestinalis* oocysts and treated with diclazuril 0.05 mg/Kg body weight orally for three consecutive days beginning from the 4th day post coccidiosis challenge): (**A**) showing hyperplasia of the intestinal mucosa; (**B**) showing moderate infestation with the developmental stages of *E. intestinalis* in the intestinal mucosa (arrow). (**C**,**D**) Extract-treated group (challenged with 3 × 10^4^ *E. intestinalis* oocysts and treated with *F. sycomorus* extract 100 mg/Kg body weight orally for four consecutive days beginning from the 4th day post challenge): (**C**) showing hyperplasia of the intestinal mucosa; (**D**) showing mild infestation with the developmental *E. intestinalis* in the intestinal mucosa (arrow). HE stain, (**A**,**C**) = ×4; (**B**,**D**) = ×20.

Histopathological investigation of the intestinal tissues of the *E. intestinalis*-infected groups revealed a marked hyperplasia in the intestinal mucosa along with severe infection with different developmental stages of coccidia parasite (Figure 3C,D). In the *E. intestinalis*-infected and treated with diclazuril group, the histopathological investigation reveals hyperplasia of the intestinal mucosa with a moderate infection with different developmental stages of coccidia parasite (Figure 4A,B). On the other hand, in the *E. intestinalis*-infected group treated with *F. sycomorus* extract, the histopathological examination showed hyperplasia of the intestinal mucosa with a mild infection with different developmental stages of coccidia parasite (Figure 4C,D).

**Figure 5:**
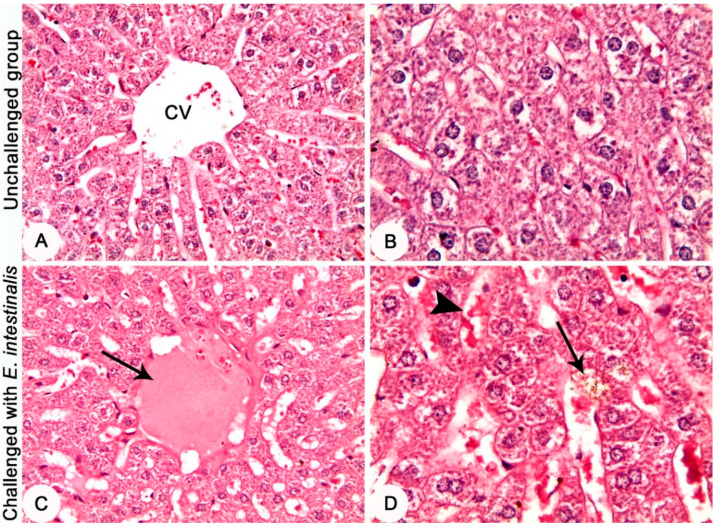
Liver of rabbits at the 8th day post challenge with *E. intestinalis*. (**A**,**B**) Negative control group (unchallenged and untreated) showing normal histological architecture (CV, central vein). (**C**,**D**) Positive control group (challenged with 3 × 10^4^ *E. intestinalis* oocysts and untreated): (**C**) showing eosinophilic material filling the central vein (arrow); (**D**) showing dilatation of bile canaliculi which engorged with bile (arrow) and dilatation and engorgement of blood sinusoids (arrowhead). HE stain, (**A**,**C**) = ×20; (**B**,**D**) = ×40.

**Figure 6:**
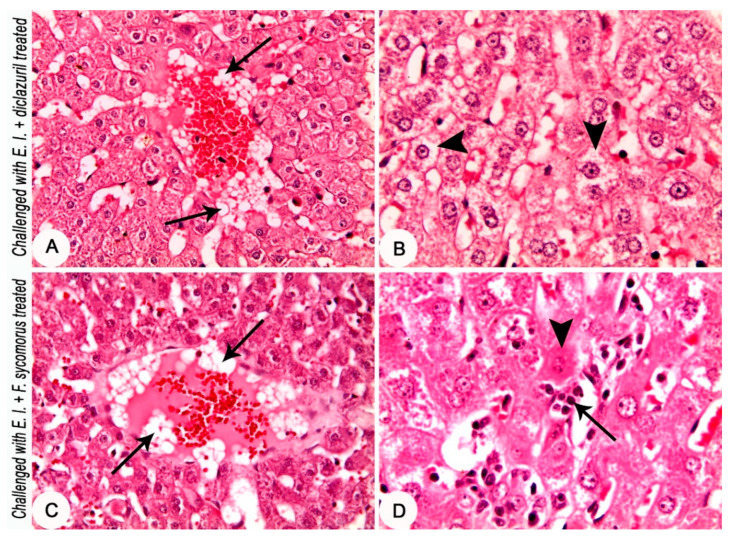
Liver of rabbits at the 8th day post challenge with *E. intestinalis*. (**A**,**B**) Diclazuril-treated group (challenged with 3 × 10^4^ *E. intestinalis* oocysts and treated with diclazuril 0.05 mg/Kg body weight orally for three consecutive days beginning from the 4th day post challenge): (**A**) showing aggregation of a large number of fat globules in the bloodstream of central vein (arrows); (**B**) showing moderate hydropic degeneration and swelling of hepatocytes (arrowheads). (**C**,**D**) Extract-treated group (challenged with 3 × 10^4^ *E. intestinalis* oocysts and treated with *F. sycomorus* extract 100 mg/Kg BW orally for four consecutive days beginning from the 4th day post challenge): (**C**) showing aggregation of a large number of fat globules in the bloodstream of central vein (arrows); (**D**) showing slight coagulative necrosis of some hepatocytes (arrowhead) and proliferation of Kupffer cells (arrow). HE stain, (**A**,**C**) = ×20; (**B**,**D**) = ×40.

Histopathological investigation of the hepatic tissues of the *E. intestinalis*-infected groups revealed an eosinophilic material in the central vein; dilatation of bile canaliculi that was engorged with bile; and dilatation and engorgement of the blood sinusoids (Figure 5C,D). The *E. intestinalis*-infected group treated with diclazuril showed an aggregation of a large number of fat globules in the bloodstream of the central vein; and the hepatocytes suffered from a moderate hydropic degeneration (Figure 6A,B). The *E. intestinalis*-infected group treated with *F. sycomorus* extract showed an aggregation of a large number of fat globules in the bloodstream of the central vein. Additionally, slight coagulative necrosis of some hepatocytes and proliferation of Kupffer cells was detected (Figure 6C,D).

### 3.5. Impacts of F. sycomorus Extract on the Growth Performance

A more significant decrease in the covariate final body weight was reported in the positive control group (2386.25 ± 63.72 g), diclazuril-treated (2386.67 ± 54.81 g), or extract-treated (2371.25 ± 58.46 g) groups than the negative control group (2568.33 ± 72.17 g). However, the extract-treated group recorded a nonsignificant decrease in covariate final body weight compared with the diclazuril-treated group. The same trends were reported in body weight gain and feed intake results (*p* < 0.01). In contrast, the feed conversion ratio was higher in the challenged groups compared with the negative control group (10.89 ± 1.67). Additionally, the feed conversion ratio was higher in diclazuril (21.07 ± 5.19) and extract (26.89 ± 7.53) treated groups compared with the control negative group (*p* < 0.05). The growth performance parameters of different experimental groups during 16 days post challenge are shown in Table 6.

## 4. Discussion

Rabbits are extremely sensitive to enteric pathogens, especially during the early weaning stage. This high sensitivity could be due to the unestablished intestinal microbiota, underdeveloped digestive system, and alteration in the gut pH [7]. Currently, several alternative bioactive substances are used successfully instead of antibiotics for controlling of coccidiosis [42]. *F. sycomorus* is one of the unique plants exhibiting antibacterial, antifungal, and antiprotozoal activities. The current work was conducted to examine the effect of *F. sycomorus* extract on rabbits infected with *E. intestinalis*. Our findings revealed successful control of the *E. intestinalis* oocyst output via oral use of the methanolic stem bark extract of *F. sycomorus* for the experimentally infected rabbits. In addition, the histopathological investigation of the intestinal tissues revealed that both diclazuril and *F. sycomorus* extract may exhibit anticoccidial effects against *E. intestinalis*. These results could be due to the effect of chlorogenic acid as an anticoccidial, antioxidant, anti-inflammatory and the improvement of intestinal barrier function [21] and the antiprotozoal effect of quercetin [18]. Both chlorogenic acid and quercetin were detected in the *F. sycomorus* methanolic extract [17,18,19]. *F. sycomorus* stem bark is a rich source of quercetin, which belongs to procyanidin tannins [17]. This flavonoid has a patient antimicrobial effect [43]. In addition, procyanidin A1 was detected in the methanolic extract of the *F. sycomorus* stem bark [19]. Procyanidin A has efficient antimicrobial effects and antibiofilm properties against antibiotic-resistant bacteria [44]. Moreover, the presence of biochanin A in the methanolic extract of *F. sycomorus* stem bark [19] could have a great role in inhibition of other gut bacteria and preventing secondary infection, especially with clostridia, which may aggravate during the coccidiosis course. Biochanin exhibit antimicrobial property in vitro against all gut clostridia [45]. 

Furthermore, the antiprotozoal nature of the *F. sycomorus* stem bark extract could be due to the presence of ficin, a proteolytic enzyme extracted from fig tree latex (*Ficus carica*) [46]. This proteolytic enzyme could be used to destroy staphylococcal biofilm due to its potential antimicrobial nature and improve the effectiveness of some antibiotics via disruption of biofilm matrices of biofilms-embedded cells [47]. Moreover, ficin had efficient anthelmintic activity [48]. Additionally, *F. sycomorus* extract contains a high content of tannins [17], which hasten the healing of inflammation of the mucous membrane [49]. Similar antiprotozoal effects were reported for *F. sycomorus* extracts in previous research; for example, trypanocidal [28]. Legendre et al. reported a significant reduction in the *Eimeria* oocyst output by 60% in weaned rabbits fed a ration containing 34% dehydrated sainfoin pellets as a source of tannins [16].

Indeed, colonization of any intestinal parasite may affect the gut microbiome [50]. Coccidiosis is associated with dysbiosis and disruption of intestinal immunity [51]. It was reported that flavonoids may modulate the gut microbiome towards the positive side [52], which might help in controlling coccidiosis. In addition, the presence of eriodictyol in the extract [19], with its anti-oxidant and anti-inflammatory effect [53], could decrease intestinal tissue inflammation and facilitate rapid healing. This may explain the reduction of intestinal histopathological lesions in rabbits infected with coccidia and treated with *F. sycomorus* compared with the positive control group. *F. sycomorus* methanolic extract showed significantly lower oocysts output compared with diclazuril-treated group. This could be due to the fact that the majority of coccidiostats such as diclazuril rarely affect coccidiosis in rabbits after the appearance of its clinical signs [54]. Consequently, these drugs are greatly effective when used during the first day of oocysts exposure [55]. The effectiveness of the anticoccidial compounds in the treatment of coccidiosis maybe associated with the increasing or decreasing of the clinical signs and lesions [55]. 

The hemogram results revealed that positive control, diclazuril-, and extract-treated groups had similar erythrocyte count and Hb concentration compared with the negative control group. In contrast, Petrova et al. reported a significant decrease in RBCs and Hb values in *Eimeria*-infected rabbits, which could be due to liver damage and subsequent inflammation because of the infection [56]. The outcomes of this study also suggested a significant increase in the total leukocyte count in the diclazuril- and extract-treated groups, along with a significant decrease in lymphocyte count. The findings matched those of Braide et al. [57], as these results could be due to the inflammatory immune response induced by *E. intestinalis*. Cam et al. reported a similar finding and assumed the effects to be a result of an inflammatory response to infection [58]. Similarly, Freitas et al. reported a significant leukocytosis in rabbits infected with *Eimeria stiedae* [59]. Al-Taee and Al-Zubaidi [60] revealed that the immunity against intracellular microorganisms is mainly a cell-mediated type of immunity. The higher percentage of the granulocytes in the infected rabbits could be due to the higher activity of the granulocyte–macrophage colony-stimulating factor, which stimulates the bone marrow stem cells to produce more granulocytes, including eosinophils [60]. Furthermore, a significant decrease in serum ALT was observed in the extract-treated group, which is in agreement with other studies [30,61].

The previously published studies suggested that the extract of *F. sycomorus* has a hepatoprotective effect; however, there is a lack of histopathological evidence in these studies [30,62]. Notably, our histopathological results were opposite to these findings. The extract-treated group showed an aggregation of numerous fat globules in the bloodstream of the central vein. In addition, slight coagulative necrosis along with a proliferation of Kupffer cells was also observed inside the hepatic tissue. Such histopathological features were absent in the other experimental groups. The histopathological feature of the hepatic tissues evidenced slight coagulative necrosis in the *F. sycomorus* extract-treated group. These findings could be due to the hepatotoxic effect of *F. sycomorus* stem bark extract on rabbit hepatic tissues. Dawod and others found coagulative necrosis with Kupffer cell proliferation in the hepatic tissue in rabbits treated orally with methanolic extract of the *F. sycomorus* stem bark, and this effect was dose-dependent [19]. In contrast, El-Sayyad et al. reported a significant hepatoprotective activity of the *F. sycomorus* extract against N-nitrosodiethylamine (NDEA) and carbon tetrachloride (CCl4)-induced hepatocarcinogenesis in rats [30]. Additionally, a marked aggregation of fat globules was observed in the bloodstream of the central vein of the liver in the *F. sycomorus*-treated group. Similarly, Dawod et al. found a marked aggregation of the fat globules in the hepatic central veins in rabbits supplemented with *F. sycomorus* stem bark extract at a dose of 100 mg/Kg for 60 days [19]. These effects could be due to the hypolipidemic nature of *F. sycomorus* extract [19,44,49]. Furthermore, Oyewole et al. found a significant increase in cholesterol and triglycerides levels in hepatic tissues of rats supplemented with leaf extract of *Ficus exasperate*. However, these compounds were to be found significantly lower in blood. This could explain the presence of the fat droplets in the central vein of the liver tissue due to the generation of these compounds within the hepatic tissues to compensate their shortage in the serum [63]. On the other hand, the histopathologic investigation revealed moderate hydropic degeneration in hepatic tissues of the diclazuril-treated group. This effect could be due to the toxic effect of diclazuril on the liver tissue. In contrast, minimal or no histopathological changes in the liver of diclazuril-treated rabbits were reported [40,56,64].

The *F. sycomorus*-treated group showed a non-significant increase in the covariate final body weight, body gain, and feed intake as well as a significant decrease in the feed conversion rate compared with the control positive group. This could be attributed to the positive effect of *F. sycomorus* extract in terms of reduction of oocyst shedding, anti-oxidant, and anti-inflammatory effects. Pakandl recorded that the assessment of weight gains is still the best precise tool to evaluate the strength of coccidiosis infection in rabbits during their growing phase [54]. The non-significant decrease in body weight in the extract-treated rabbits compared with the diclazuril-treated group could be due to the anti-nutritional effect of the *F. sycomorus* tannins, as the *F. sycomorus* stem bark is rich in condensed tannins (procyanidin type) [17]. These tannins could bind to the intake proteins and change their physicochemical nature. In addition, it has an astringent nature that may adversely affect food palatability, food intake, and growth performance [49]. Although tannins have been traditionally considered antinutritional factors, they may be beneficial for animals. Redondo et al. reported that the chemical structure of tannins (associated with the plant origin), the final concentration in feed, the physiological state of animals, and the composition of the diet are detrimental factors for these beneficial effects on the digestive function of birds [49]. Therefore, the global health has to be analyzed and assessed before the extract is used as a feed additive or complement.

## 5. Conclusions

The stem bark methanolic extract of *F. sycomorus* exhibited anticoccidial effects against *E. intestinalis* infection in rabbits despite the adverse effects on the hepatic tissue and growth performance. Further research may be needed to optimize the method of extraction and the dose of the extract for an efficient anticoccidial effect with minimal adverse health events. Additionally, the mode of action of *F. sycomorus* needs to be investigated.

## Figures and Tables

**Table 1:** Feed composition of the experimental diet for 3 weeks growing period.

Ingredients	Composition (%)
Maize	40.00
Soybean meal	10.00
Dried alfalfa	20.00
Rice bran	10.00
White bran	14.00
Fish meal (72% CP)	2.00
Bone meal	2.00
Limestone	1.00
Premix (Growers) ^1^	0.50
Salt (NaCl)	0.50
Total	100.00
Calculated nutrients	
Crude protein (%)	16.73
Crude protein	16.73
Crude fiber	9.20
Calcium	1.25
Available phosphorus	0.40
Sodium	0.24
Apparent metabolizable energy (AME; Kcal/kg)	2600
Digestible methionine	0.41
Digestible lysine	1.27
Digestible threonine	0.73
Choline	0.75
Digestible total sulfur amino acids (TSAA)	0.84

^1^ The premix provided per kilogram of diet: vitamin A (retinyl acetate), 12,000 IU; cholecalciferol, 270 IU; vitamin E (DL-α-tocopheryl acetate), 44 IU; vitamin K, 4.0 mg; thiamine mononitrate (B1), 8.80 mg; riboflavin (B2), 17.60 mg; pyridoxine HCL (B6), 8.80 mg; vitamin B12 (cobalamin), 26.40 mg; D-pantothenic acid, 15 mg; folic acid, 2.20 mg; niacin, 176.00 mg; biotin, 0.44 mg; choline 0.88 mg/kg; Mn (MnSO_4_H_2_O), 150.00 mg/kg120 mg; Cu, 60.00 mg; Zn, 120.00 mg/kg 100 mg, Se, 0.60 mg/kg 0.3 mg; Fe (FeSO_4_·7H_2_O), 50 mg.

**Table 2:** Experimental design for evaluation of the anticoccidial effect of *F. sycomorus*.

Group No.	Number	Challenge with *E. intestinalis*		Treatment		Assessment Parameters
		Age	Dose	Drug	Dose	
1	10	-	-	-	-	1—Oocyst shedding 2—Clinical symptom 3—Hematological parameters 4—Histopathological findings 5—Growth performance
2	10	10 weeks	3 × 10^4^	-	-	
3	10	10 weeks	3 × 10^4^	Diclazuril 10%	0.05 mg/kg	
4	10	10 weeks	3 × 10^4^	*F. sycomorus*	100 mg/Kg	

**Table 3:** Effects of treatment (unchallenged and untreated; challenged with *E. intestinalis* and untreated; challenged with *E. intestinalis* and treated with diclazuril; and challenged with *E. intestinalis* and treated with *F. sycomorus* extract) on the clinical signs exhibited in rabbits at the 8th day post challenge.

Clinical Signs	No. of Animals	Control − ve ^1^		Control + ve ^2^		Diclazuril 0.05 mg/kg ^3^		Extract 100 mg/kg ^4^		*p*-Value ^5^
		Freq.	%	Freq.	%	Freq.	%	Freq.	%	
Depression	10	0	0	3	30	2	20	2	20	0.46
Diarrhea	10	0	0	5	50	2	20	0	0	0.01

^1^ Negative control group: unchallenged and untreated. ^2^ Positive control group: challenged with 3 × 10^4^ *E. intestinalis* oocysts and untreated. ^3^ Diclazuril-treated group: challenged with 3 × 10^4^ *E. intestinalis* oocysts and treated with diclazuril 0.05 mg/kg BW orally for three consecutive days beginning from the 4th day post challenge. ^4^ Extract-treated group: challenged with 3 × 10^4^ *E. intestinalis* oocysts and treated with *F. sycomorus* extract 100 mg/kg body weight orally for four consecutive days beginning from the 4th day post coccidiosis challenge. ^5^ Fisher’s exact test, significant at *p* < 0.05.

**Table 4:** Effects of treatment (unchallenged and untreated; challenged with *E. intestinalis* and untreated; challenged with *E. intestinalis* and treated with diclazuril; and challenged with *E. intestinalis* and treated with *F. sycomorus* extract) on the average of liver enzymes, erythrogram, and leukogram of rabbits through 16 days post challenge.

Parameters		Number	Control − ve ^1^	Control + ve ^2^	Diclazuril, 0.05 mg/kg ^3^	Extract, 100 mg/Kg ^4^	*p*-Value
Liver enzymes	ALT (U/L)	7	27.26 ± 3.55 ^b^	34.01 ± 3.55 ^a^	21.10 ± 3.27 ^b^	20.17 ± 3.29 ^b^	0.03
	AST (U/L)	7	16.44 ± 3.50	13.67 ± 3.39	18.40 ± 3.22	12.22 ± 3.25	0.18
	ALP (IU/L)	7	80.49 ± 11.76	106.67 ± 12.99	94.87 ± 11.76	88.13 ± 14.71	0.56
Erythrogram	RBCs (×10^6^)	7	5.64 ± 0.06	5.44 ± 0.06	5.49 ± 0.06	5.47 ± 0.06	0.98
	Hb (g/dL)	7	12.46 ± 0.28	11.92 ± 0.29	12.18 ± 0.25	12.18 ± 0.26	0.45
	MCV (fL)	7	43.81 ± 0.63 ^a^	41.28 ± 0.64 ^b^	40.88 ± 0.56 ^b^	40.90 ± 0.58 ^b^	0.03
	MCH (pg)	7	22.02 ± 0.26	21.85 ± 0.27	22.15 ± 0.24	22.23 ± 0.25	0.19
	MCHC (%)	7	51.75 ± 1.09 ^b^	54.40 ± 1.12 ^ab^	56.11 ± 0.98 ^a^	56.18 ± 1.02 ^a^	0.05
Leukogram	WBCs (×10^3^)	7	7.81 ± 0.03 ^c^	9.80 ± 0.03 ^b^	10.21 ± 0.02 ^a^	9.70 ± 0.03 ^b^	0.05
	Lymphocytes %	7	70.34 ± 2.71 ^a^	53.83 ± 2.78 ^c^	61.76 ± 2.44 ^b^	55.06 ± 2.52 ^bc^	0.05
	Monocytes %	7	10.11 ± 0.73 ^a^	10.49 ± 0.73 ^a^	9.25 ± 0.73 ^c^	9.37 ± 0.73 ^b^	0.04
	Granulocytes%	7	25.83 ± 2.34 ^c^	39.79 ± 2.40 ^a^	33.97 ± 2.10 ^b^	39.74 ± 2.17 ^a^	0.03

^1^ Negative control group: unchallenged and untreated. ^2^ Positive control group: challenged with 3 × 10^4^ *E. intestinalis* oocysts and untreated. ^3^ Diclazuril-treated group: challenged with 3 × 10^4^ *E. intestinalis* oocysts and treated with diclazuril 0.05 mg/Kg BW orally for three consecutive days beginning from the 4th day post challenge. ^4^ Extract-treated group: challenged with 3 × 10^4^ *E. intestinalis* oocysts and treated with *F. sycomorus* extract 100 mg/Kg body weight orally for four consecutive days beginning from the 4th day post coccidiosis challenge. ALT, alanine aminotransferase; AST, aspartate aminotransferase; ALP, alkaline phosphatase; RBCS, red blood cells; MCV, mean corpuscular volume; MCH, mean corpuscular hemoglobin; MCHC, mean corpuscular hemoglobin concentration; WBCs, white blood cells. Values are presented as least square mean ± SE. ^a–c^ Means within the same row with different superscripts are statistically different at *p* < 0.05 (repeated measure ANOVA, Bonferroni post hoc test).

**Table 5:** Effects of treatment (unchallenged and untreated; challenged with *E. intestinalis* and untreated; challenged with *E. intestinalis* and treated with diclazuril; and challenged with *E. intestinalis* and treated with *F. sycomorus* extract) on *E. intestinalis* lesion scores in the jejunum at the 8th day post challenge.

Lesions	No.	Lesion Score ^1^				Chi-Square	*p*-Value
		Control − ve ^2^	Control + ve ^3^	Diclazuril, 0.05 mg/kg ^4^	Extract, 100 mg/kg ^5^		
Intestine							
Mucosal hyperplasia	3	0.00 ± 0.00 ^b^	3.33 ± 0.33 ^a^	2.83 ± 0.17 ^b^	3.00 ± 0.00 ^b^	8.92	0.03
The intensity of coccidia parasite in the intestinal mucosa	3	0.00 ± 0.00 ^b^	4.00 ± 0.00 ^a^	3.17 ± 0.17 ^b^	1.83 ± 0.17 ^b^	10.76	0.01
Liver							
Hydropic degeneration of hepatocytes	3	0.00 ± 0.00 ^b^	0.00 ± 0.00 ^b^	2.83 ± 0.17 ^a^	1.00 ± 0.29 ^ab^	10.87	0.01
Coagulative necrosis of hepatocytes	3	0.00 ± 0.00 ^b^	0.00 ± 0.00 ^b^	0.00 ± 0.00 ^b^	0.83 ± 0.17 ^a^	10.80	0.01
Dilatation and engorgement of blood sinusoids	3	0.00 ± 0.00 ^b^	2.83 ± 0.17 ^a^	1.83 ± 0.17 ^ab^	1.00 ± 0.00 ^ab^	10.76	0.01
Dilatation and engorgement of bile duct	3	0.00 ± 0.00 ^b^	1.67 ± 0.33 ^a^	0.00 ± 0.00 ^b^	0.00 ± 0.00 ^b^	10.80	0.01
Presence of fat globules in the bloodstream of central veins	3	0.00 ± 0.00 ^b^	2.33 ± 0.33 ^b^	4.00 ± 0.00 ^a^	4.00 ± 0.00 ^a^	10.87	0.01

^1^*Eimeria intestinalis* lesion score in the jejunum at the 8th day post challenge. ^2^ Negative control group: unchallenged and untreated. ^3^ Positive control group: challenged with 3 × 10^4^ *E. intestinalis* oocysts and untreated. ^4^ Diclazuril-treated group: challenged with 3 × 10^4^ *E. intestinalis* oocysts and treated with diclazuril 0.05 mg/Kg BW orally for three consecutive days beginning from the 4th day post challenge. ^5^ Extract-treated group: challenged with 3 × 10^4^ *E. intestinalis* oocysts and treated with *F. sycomorus* extract 100 mg/Kg body weight orally for four consecutive days beginning from the 4th day post coccidiosis challenge. *p*-value (Fisher’s exact test, significant at *p* < 0.05). The 0, 1, 2, 3, and 4 scores denoted absence, minimal, mild, moderate, and severe lesions, respectively. Values are presented as mean ± SE. ^a,b^ Means within the same row with different superscripts are statistically different at *p* < 0.05 (Kruskal–Wallis one-way analysis of variance with Dunn–Bonferroni post hoc test).

**Table 6:** Effects of treatment (unchallenged and untreated; challenged with *E. intestinalis* and untreated; challenged with *E. intestinalis* and treated with diclazuril; and challenged with *E. intestinalis* and treated with *F. sycomorus* extract) on initial body weight, covariate final body weight, body gain, feed intake, and feed conversion of rabbits through 16 days post challenge.

Group	No. of Animals	Initial Body Weight	Covariate Final Body Weight (g)	Body Gain (g)	Feed Intake (g)	Feed Conversion
Control − ve ^1^	7	2410.33 ± 52.26	2568.33 ± 72.17 ^a^	158.00 ± 21.82 ^a^	1523.33 ± 104.91 ^a^	10.89 ± 1.67 ^c^
Control + ve ^2^	7	2340.00 ± 46.51	2386.25 ± 63.72 ^b^	46.25 ± 20.66 ^b^	971.50 ± 77.93 ^b^	38.97 ± 8.39 ^a^
Diclazuril-treated, 0.05 mg/kg ^3^	7	2290.67 ± 37.60	2386.67 ± 54.81 ^b^	96.00 ± 25.53 ^ab^	1367.67 ± 82.68 ^ab^	21.07 ± 5.19 ^b^
Extract-treated 100 mg/kg ^4^	7	2311.00 ± 52.64	2371.25 ± 58.46 ^b^	60.25 ± 14.67 ^b^	1067.38 ± 129.78 ^b^	26.89 ± 7.53 ^b^
*p*-value		0.39	0.01	0.01	0.01	0.05

^1^ Negative control group: unchallenged and untreated. ^2^ Positive control group: challenged with 3 × 10^4^ *E. intestinalis* oocysts and untreated. ^3^ Diclazuril-treated group: challenged with 3 × 10^4^ *E. intestinalis* oocysts and treated with diclazuril 0.05 mg/Kg BW orally for three consecutive days beginning from the 4th day post challenge. ^4^ Extract-treated group: challenged with 3 × 10^4^ *E. intestinalis* oocysts and treated with *F. sycomorus* extract 100 mg/Kg body weight orally for four consecutive days beginning from the 4th day post coccidiosis challenge. Values are presented as mean ± SE. ^a–c^ Means within the same column with different superscripts are statistically different at *p* < 0.05 (one way ANOVA, Duncan’s post hoc test).

## Data Availability

Not applicable.
